# Length is not all that matters: testing the role of number identity and the ratio of fillers in comparisons of multi-digits with different digit length

**DOI:** 10.1007/s00426-022-01655-1

**Published:** 2022-02-18

**Authors:** Javier García-Orza, Ismael Gutiérrez-Cordero, Carlos Larios, Anikó Csilinkó, Juan Antonio Álvarez-Montesinos

**Affiliations:** 1 Numerical Cognition Lab, Facultad de Psicología, Universidad de Málaga, Campus de Teatinos, s/n, 29071 Málaga, Spain; 2 Instituto de Investigación Biomédica de Málaga, Málaga, Spain

## Abstract

Research in multi-digit number comparison usually considers stimuli with the same number of digits (e.g., 3452 vs. 7831). Surprisingly, there is almost no research on the comparison of numbers that differ in length (e.g., 995 vs. 1000), which demands a focus on the number of digits in each multi-digit, despite the fact that the role of number length has been explicitly acknowledged in componential models of multi-digit processing. Our study explores whether the comparison of pairs of natural numbers that differ in length is affected by the identity of the leftmost digit of each multi-digit, and asks what is the effect of having variable proportions of trials with pairs of numbers of the same-length in the task. Across three studies participants compared numbers in blocks with different proportions of same-length multi-digit pairs (Experiment 1 and 2: 25% vs. 50% vs. 75%; Experiment 3: 0% vs. 50%). Stimuli in the different-length condition were length-digit congruent (the number with more digits starting with a larger digit: 2384 vs. 107) or length-digit incongruent (the number with more digits starting with a smaller number: 2675 vs. 398). Response times were shorter in length-digit congruent pairs than in the incongruent pairs. Unexpectedly, this effect was only slightly modulated by the proportion of same-/different-length multi-digit pairs in the experimental set. Despite its perceptual saliency, length is not the only information considered when comparing different-length numbers. The leftmost-digit is also taken into account, with variable relevance here, depending on the characteristics of the stimuli set.

## Introduction

Advertisers have long known the impact that length, in terms of the number of digits, has on the comparison of prices. Thus, promotions such as the following (fictitious) one are familiar around the world: “Was $1000, now only $999”. Despite this, not much is known in the field of numerical cognition about the comparison of multi-digits (i.e., numbers composed of more than one digit) that differ in length (i.e., in a number of digits: e.g., 456 vs. 2398). The absence of studies on the comparison of numbers of different lengths is probably due to the fact that differences in the length of the number strings are so evident and so determinant in the comparison process (when comparing two natural numbers the one with more digits represents a larger quantity, independently of the identity of the constituent digits) than once detected, any remaining information, such as the identity of the digits themselves, is irrelevant. Some recent accounts of numerical comparison, though, go against this view, and as far as we know no direct results regarding this issue with natural numbers are available since it is yet to be addressed. The aim of our current study is to understand how multi-digit numbers of different lengths are compared.

Numerous studies have been conducted to examine the nature of processing Arabic multi-digit numbers, and these findings demonstrate the complexity of the process: it consists of identifying and coding the relative position of each digit, activating the place-value of each, plus a final voluntary process of place-value computation (Cipora et al., 2019; García-Orza & Damas, 2011; García-Orza & Perea, 2011; García-Orza et al., 2017; Kallai & Tzelgov, 2012; Korvorst & Damian, 2008; Meyerhoff et al., 2012; Nuerk et al., 2001).

To investigate the processing of multi-digit numbers, comparison tasks have mainly been used (although to observe the use of other methods with exceptionally large numbers refer to e.g., Landy et al., 2017). In these tasks, pairs of numbers are presented and participants are requested to press a key in deciding which is the larger (or the smaller) number. Although details vary regarding the specific demands of the task (e.g., comparing natural, negative or decimal numbers; using a reference or presenting pairs of numbers simultaneously or sequentially) and the configuration of the stimuli set (e.g., the proportion of experimental trials vs. fillers, compatible vs. incompatible pairs), there is a large body of evidence indicating that different attributes are taken into account before reaching a decision (see Huber et al., 2016, for a review).

For instance, in the comparison of same-length natural numbers (e.g., 345 vs. 671) the identity of the leftmost digit number is the most relevant factor, but responses also seem to be influenced by the identity of the other digits (e.g., Korvorst & Damian, 2008; Nuerk et al., 2001; Poltrock & Schwartz, 1984). Of particular interest here is evidence from the unit-decade compatibility effect (Nuerk et al., 2001), which shows slower response times when comparing pairs of two-digit numbers in which the unit digit is larger than the smaller number (e.g., 47 vs. 92) compared to pairs in which the unit digit is larger than the larger number (e.g., 42 vs. 87). This compatibility effect clearly shows that multi-digit number comparison does not proceed exactly in a “step by step” fashion, but in parallel and interactively, because the comparison process is applied to units despite the leftmost digits of the numbers to be compared (in this case, those in the decades) are clearly different. This effect has been also shown in three-digit numbers (e.g., Korvorst & Damian, 2008; Bahnmueller et al., 2015, 2016, 2021) and in some cases in four- and six-digit numbers, although in these cases a combination of sequential and parallel processes has been observed (e.g., Meyerhoff et al., 2012).

Similarly, when comparing positive and negative numbers (e.g., − 57 vs. + 93) the polarity is the most relevant factor; however, number identity has also been observed to influence decisions (e.g., Ganor-Stern & Tzelgov, 2008; Huber et al., 2015; Krajcsi & Igács, 2010; Tzelgov et al., 2009; Zhang et al., 2018). So, it takes longer to respond to sign-digit compatible pairs like − 21 vs. + 46 than to incompatible pairs like − 46 vs. + 21, thus evidencing a sign-digit compatibility effect (Huber et al., 2016).

Interestingly for the present study, the role of length has been shown to be relevant in the comparison of decimal numbers (e.g., 0.23 vs. 0.7). In this case, the identity of the leftmost decimal is the most important factor, but the length in the decimal part of the number also seems to affect responses (e.g., see Cohen, 2010; Huber et al., 2014; Kallai & Tzelgov, 2014; Varma & Karl, 2013). Thus, a so-called string-length congruity effect exists, in which people respond faster to congruent trials like 0.2 vs. 0.53 than to trials like 0.23 vs. 0.5, where the smaller number has more digits in its decimal part (Huber et al., 2014).

These effects support a componential view of number processing and the idea of comparison as a process where different attributes are considered in parallel (Huber et al., 2016, see below). Applied to the comparison of natural numbers of different lengths, this view would predict that although length is the most relevant factor in the task, responses may be affected by other factors, in this case, the identity of the leftmost digit. So, it seems reasonable to expect that pairs like 2384 vs. 107, in which the number with more digits starts with a larger digit (i.e., length-digit congruent), are processed easier than pairs like 2675 vs. 398, where the number with more digits starts with a smaller number (i.e., length-digit incongruent), thus giving rise to a length-digit congruity effect^1^.

Another factor of interest to research into number comparison is related to the characteristics of the items set. The modulation of experimental effects by set composition is usual in psychological tasks. For example, it is well known that the interference in the Stroop effect decreases as incongruent trials become more frequent (Tzelgov et al., 1992). In the case of number comparison it has also been shown that participants adapt their responses to the nature of the items presented, so that when comparing trials composed of a positive and a negative number the focus is mainly on the + and − signs and the influence of the sign-digit compatibility effect is relatively small; however, when filler items with either positive or negative numbers are included, the relevance of the leftmost digit increases and then the sign-digit compatibility effect is seen more clearly (Huber et al., 2015). The items set composition also affects the unit-decade compatibility effect. Macizo and Herrera (2011, see also Moeller et al., 2011) presented participants with pairs of two-digit word numbers and manipulated the ratios of same-/different-decade pairs (20%, 50%, 70%). They found that the relative relevance of the units was modulated by the items set: the more same-decade pairs, the more relevance was attributed to the units, even those in different decade pairs, and this increased the compatibility effect. Another factor that seems to modulate the unit-decade compatibility effect is the proportion of compatible/incompatible items (e.g., Huber et al., 2013; Macizo & Herrera, 2013). So, as the percentage of incompatible trials increased in the items set, a decrease in the compatibility effect was observed. All these results on the effect of the composition of the items set have been interpreted as evidence of the modulation of the numerical effects described above by a cognitive control system that monitors participants’ responses. This cognitive control mechanism seems to act directly over the process that weighs the influence of each parameter (e.g., leftmost digit, sign, length) in the comparison task.

The relevance of the different attributes of the numbers in multi-digit comparison tasks, as well as those related to the conditions of the task, has been included in a recent computational model by Huber et al. (2016). This model conceives of multi-digit comparison as a componential process in which different attributes (polarity signs, length and digits, e.g., units, tens, hundreds, etc.) are processed in parallel and weighted by their relevance for the task at hand. In this computational model four layers are distinguished (see Huber et al., 2016, Fig. 2). In the input layer, the number stimuli are processed, and the polarity sign, the magnitude of the digits (e.g., hundreds, tenths and units digits), and the number of digits in each number are registered by different networks working in parallel. In the comparison layer, these types of information are contrasted. The activation of task demand nodes and the connection weights between the comparison and the response layers determine the relevance of each parameter (hundreds, length, polarity), meaning that depending on the task demands more weight is assigned to the corresponding comparison node. For instance, in the comparison between three-digit numbers larger activation is assigned to the hundred digits, whereas in the comparison between numbers of different lengths more activation is probably given to the length node by the task demand layer. The model also incorporates a cognitive control network that modulates the activation of the task demand nodes and that, hence, makes the system sensitive to variations in the stimuli set and allows the system to monitor error detection (Huber et al., 2016). So, in cases of having a task with a high proportion of numbers with the same leftmost digit (e.g., 76 vs. 79) more weight is given to the units, whereas in the case of a low proportion of these numbers less weight is given to the units and more to the decades.

From the viewpoint of this componential model, it is predicted that when comparing numbers of different length, despite length being the relevant information to decide which number is larger, other attributes processed in parallel, such as the identity of the leftmost digit, may affect the comparison process, even though they have a secondary role in the task. This secondary role may vary depending on the characteristics of the stimuli set, so if same-length numbers are added to a list with different-length numbers, the value of the leftmost digits will gain relevance since, in same-length trials, this is the relevant information to identify the larger number. Although the model posits a dedicated mechanism to analyze length together with the rest of the attributes during the comparison process, there is currently no empirical data on the issue of using natural numbers. Our aim is to prove such data.

Despite the experimental support for the model, there is, however, some data that does not support a componential and parallel view of numerical comparison. Poltrock and Schwartz (1984) found evidence of sequential processing in the comparison of four- and six-digit numbers. Response times increased with the position of the differing numbers, so the rightmost in the string the numbers that differ are, the larger the response latency^2^. Similarly, in an eye-tracking experiment, Meyerhoff et al. (2012) found unit-decade compatibility effects in two-digit numbers but these effects were not observed in four-digit numbers and evidence of both sequential and parallel processing was observed in six-digit numbers. In particular, in four- and six-digit numbers, participants RT and fixation pattern evidenced a sequential processing of digits from left to right: RTs increased the more to the right the position of the differing numbers in the pair was; and fixations going from left to right did not continue once differing numbers were found. Compatibility effects were found in six-digit numbers but only when the compatible/incompatible number pairs were at the beginning of the digit string. So, these data show that parallel processing is not always the rule, but rather it depends on characteristics of the stimuli: parallel processing is clear for two-digit numbers, is not clear for four-digit numbers, and is combined with serial processes in six-digit numbers (Meyerhoff et al., 2012). This suggests that in more complex tasks like the comparison of three and four-digit numbers, a more sequential process is feasible, this showing length to be the only factor to be considered in the task.

Other research in the area of numerical cognition has also shown that some decisions on tasks involving numbers may be taken while ignoring certain information, for instance, without relying on the meaning of numbers (Cohen, 2009; García-Orza et al., 2012; Wong & Szücs, 2013; Zhang et al., 2018). Cohen (2009) found that deciding whether a pair of single-digit numbers (e.g., 7 vs. 1) were the same or not (i.e., using a perceptual task) was explained by the perceptual similarity between the digits presented (1 and 7 are closely similar), more than by their distance on the number line as previously claimed by Dehaene and Akhavein (1995). García-Orza et al. (2012), who found similar results to Cohen, proposed a race-based approach to explain these data; they do not discount the existence of semantic processing of the numbers during the task, but, they argue, it simply takes place once a decision has been reached based on the perceptual information, thus not affecting participants’ performance in the task. On this view, it is possible that comparing different-length numbers may be solved simply by focusing on which stimuli have more digits or even the stimuli that subtend a larger representation in our retina, and hence, that the processing of other parameters like the identity of the leftmost digits does not influence the task.

To the best of our knowledge, only two studies have focused directly on the role of length in natural multi-digit numbers. In a study exploring the cost of processing place-value (e.g., Arabic) or sign-value (e. g., Roman) numerical systems, Krajcsi and Szabó (2012) asked their participants to learn a novel base-four place-value system and to subsequently compare multi-digit numbers. They found support for a fast processing of length in place-value notation. Compared to multi-digits with the same digit length, response times and errors were smaller when 2-digit numbers were compared to 3-digit numbers, and even smaller when 1-digit numbers were compared to 3-digit numbers. From these findings, they concluded that participants, when faced with different-length numbers, reach a decision by simply using a perceptual procedure and without processing the numbers.

In the other study by Hinrichs et al., (1982, Experiment 1), participants were presented with numbers from 1 to 7-digit length and asked to decide whether that number was smaller or larger than a reference: 5000. Hinrichs and cols. observed that differences in length were determinant, the greater the difference in number-length between the number and the reference, the faster the response. Interestingly, they also found a leftmost digit effect when three and five-digit length numbers were compared to 5000, the value of the overall number predicting response times here. Based on their results they rejected a serial model of multi-digit comparison. These data also support the existence of a length-digit congruity effect and thus support parallel processing of length and the identity of digits, and against length as the unique parameter to consider in the task. Importantly, their results were also compatible with a holistic model, as the response times increase with the logarithm of the difference between the reference and the number presented, and thus they cannot disentangle the issue of whether the mechanism behind the task was “converting numerical information to some magnitude (analogue) representation and making direct psychophysical comparisons or combining physical length cues (number of places) with symbolic evaluation (digit value) to determine the relative magnitude of multi-digit numbers” (Hinrichs et al., 1982, p. 495). Additionally, it is uncertain to what extent this result can be extended to the simultaneous presentation of pairs of multi-digit numbers, because the holistic representation has been observed in the comparison of two-digit numbers with the sequential presentation but decomposed with simultaneous presentation (Ganor-Stern et al., 2009, but see García-Orza & Damas, 2011; Moeller et al., 2013). Additionally, Hinrichs et al. (1982) only provide correlational evidence on the role of digit identity in the comparison of numbers of different length. Consequently, these results make it necessary to conduct further research on the relevance of digit identity in the comparison of multi-digit numbers that differ in the number of digits (i.e., in length).

## The present research

The aim of our study was to examine whether the length and the identity of the leftmost digits are processed simultaneously when comparing different-length multi-digit numbers, and how this is affected by the composition of the stimuli set. Knowing how our mind constructs these processes is a fundamental step in understanding the mechanisms implicated in building up the numerical value of multi-digit numbers. Across three studies we concentrated on the comparison of three- and four-digit numbers, as we wanted to analyze numbers within the subitizing range. Two-digits numbers were also avoided as they probably have a different status than other multi-digit numbers (Mann et al., 2012). We presented to the participants a simultaneous numerical comparison task. Experimental materials included pairs of multi-digit numbers that have the same number of digits (3 vs. 3; 4 vs. 4) or not (3 vs. 4). Stimuli in the different-length condition were length-digit congruent (the number with more digits started with a larger digit: 2384 vs. 107) or length-digit incongruent (the number with more digits started with a smaller number: 2675 vs. 398) equated in terms of overall distance (in the example, 2277). Furthermore, we manipulated the proportion of same-length pairs in the studies: blocks of 25% vs. 50% vs. 75% same-length pairs were employed in Experiments 1 & 2, and blocks of 0% vs. 50%, in Experiment 3. Additionally, in Experiment 3 we manipulated the numerical distance between the initial or leftmost digits in the comparison task, so these numbers in the congruent and incongruent condition may have a distance of 1 (e.g., 2478 vs. 103 and 2764 vs. 389) or distance 3–4 (e.g., 5598 vs. 145 and 5468 vs. 978).

With these studies, we test the predictions of the componential model proposed by Huber et al. (2016). According to this model, the value of the leftmost digits is processed in parallel with the length of numbers, hence more difficulties are expected under the length-digit incongruent condition than under the congruent condition. Furthermore, it is anticipated that the length-digit congruity effect would be modulated by the proportion of same-length fillers, in keeping with previous findings that have demonstrated that the weighting of the parameters involved in multi-digit comparison is under cognitive control (e.g., Macizo & Herrera, 2013). In same-length pairs the leftmost digits of the numbers lead the decision process, thus it is expected that increasing the number of same-length pairs will increase attention to the identity of these digits and should then maximize the length-digit congruity effect.

On the contrary, no length-digit congruity effect would be expected if the decision process used is of a serial nature that evaluates the length of each multi-digit, and solely in the case of equality then proceeds to explore the initial numbers. This would align with race-based approaches that consider that highly salient perceptual information is considered faster and hence may drive the decision process.

The scenario is also possible where, in the presence of length-digit congruity effects, there is a lack of interaction between the length-digit congruity effect and the composition of the blocks. An absence of influence of the proportion of fillers on a significant congruity effect would indicate that this effect is not under cognitive control. In other words, the relevance of the leftmost digit of a number would be significant enough to escape the cognitive control mechanism, suggesting a very automatic comparison of the leftmost digits.

In sum, our aim with these studies is to determine whether during the comparison process of different-length multi-digit numbers the length is the only factor taken into consideration.

## Experiment 1

### Method

#### Participants

Thirty-six undergraduate students from the University of Málaga took part voluntarily in this experiment. Their mean age was 22.40 (*SD* = 6.72, range 18–50 years, 9 males). An additional participant was excluded as she committed more than 20% errors. All participants had a normal or corrected-to-normal vision and were naive regarding the purpose of the study. The number of participants here was similar to those employed in previous experiments exploring the processing of multi-digit numbers (see Kallai & Tzelgov, 2012; García-Orza et al., 2017).

#### Stimuli

The experiment included four types of pairs of multi-digit numbers: two types consisted of different-length numbers (comparing three- vs. four-digit numbers) and two types were same-length numbers (comparing pairs of three- vs. three-digit numbers and four- vs. four-digit numbers). Within different-length pairs, 24 were length-digit congruent pairs where the initial (leftmost) digit of the four-digit number was larger than the initial digit of the three-digit number. Furthermore, starting from the left, digits in the second and third positions were also larger in the four-digit number than in the three-digit number (e.g., 2384 vs. 107, with 2 > 1, 3 > 0 and 8 > 7). Although compatibility effects (Nuerk et al., 2001) have not been reported in some studies using four-digit numbers (Meyerhoff et al., 2012, but see Korvorst & Damian, 2008, with three-digit numbers), this approach sought to avoid these effects (note that, if this metric place-value is instead considered, then this factor is not controlled for). In the other 24 different-length pairs, the digit-incongruent pairs, the three leftmost digits of the four-digit number were smaller than their corresponding digit in the three-digit number (e.g., 2675 vs. 398, with 2 < 3, 6 < 9 and 7 < 8). Pairs in the congruent and the incongruent trials were created in a way that the overall distance between numbers in the pair was exactly the same in both types of trials. Additionally, the difference between the leftmost digits was always one in all pairs (see an example of the stimuli in Table 1, and the complete list of stimuli in Appendix A).

**Table 1:** An example of the stimuli employed in Experiments 1 and 2

Pair type	Pair sample		Overall distance
	Bigger number	Smaller number	
Different-length numbers			
Length-digit congruent	2384	107	2277
Length-digit incongruent	2675	398	2277
Same-length numbers			
Three-digits	275	134	141
Four-digits	2147	3659	1512

In each number pair, two numbers are presented that may have the same (3 or 4) or a different number of digits (one in the pair had 3 digits and the other 4 digits) (see text for an explanation of the differences between conditions)

The remaining two types of number pairs were used as fillers and consisted of numbers of the same length, in one case the numbers to be compared were three-digit length numbers and in the other, four-digit numbers. These filler pairs were created with only one restriction: the initial numbers were always different. So, in some cases all the digits in one number were larger than in the other, whereas in other cases the leftmost digit was larger in one number but the rest, or some of the rest, were smaller. A total of 72 trials of each length were made. Overall distance between the number pairs in the same-length trials was different, being considerably smaller in the three-digit number pairs than in the four-digit number pairs, and in both cases they were smaller than in the trials of the different-length conditions (see Table 1).

Three different experimental lists were created varying the proportion of fillers. In the three lists the 48 different-length pairs (24 congruent + 24 incongruent) were included twice (once with the four-digit number on the right and once with the four-digit number on the left) for a total of 96 pairs. In the 25% same-condition list, 32 same-length pairs were added, 96 in the 50% same-condition list and 288 in the 75% same-condition list. In each list, half of the same-length pairs were always three-digit length numbers and half four-digit length numbers.

#### Procedure

Participants were tested in groups smaller than 10, under the supervision of two researchers. Each participant was allocated to a small cubicle equipped with a PC and 19-inch colour monitors. Presentation of the stimuli and recording of response times were controlled by Windows-based computers using E-prime 2.0 software (Psychology Software Tools, Pittsburgh, PA). Instruction and stimuli were presented in white on a black background using, 20 pt. Courier new. Each trial began with a fixation point (#) centered on the screen for a random time between 250 and 500 ms. The experimental stimulus consisting of two multi-digit numbers was shown next to each other at the top-center of the screen and remained there until a response had been given or 2000 ms had passed. The interval between trials was set at 800 ms. Additionally, response times were measured from the target onset until the participant’s response was detected. E-prime scripts of this and the following experiments are available on request from the corresponding author.

In a single session, the three experimental lists corresponding to the three filler ratio conditions were presented with a brief pause between them. The order of lists was counterbalanced between participants. The experimental list began with the instructions followed by 8 practice trials that had similar characteristics to those applied in the experiment. Participants were instructed to press a right or left button on the keyboard (M or Z, respectively) as accurately and quickly as possible, corresponding to the side on which the larger number appeared. During practice, feedback for accuracy was provided. After the practice trials, participants were reminded of the instructions and the experimental block began. No feedback was provided during this block. A short break was introduced after every hundredth item, to avoid respondent fatigue. Participants could decide the duration of the pause, but we suggested at the beginning that it should be no longer than 10–15 s, to which all participants agreed.

Each item was presented twice, one with the larger number on the right and the other with the larger number on the left. A total of 96 different-length trials were presented (24 pairs × 2 congruity conditions × two-sides) in each list. Same-length pairs varied in each list: 32, 96 and 288 trials for the 25%, 50% and 75% filler ratio conditions, respectively. The total number of items was 128 trials in the 25%, 192 for the 50% and 384 for the 75%^3^. The order of item presentation was randomized. The experiment, including breaks, lasted approximately 40–45 min.

### Design and analysis

The proportion of correct responses and mean response times (RTs) per participant were calculated for each condition. Same-length pairs were not analyzed as they are not related to our hypothesis, so analyses focused on the different-length pairs only. Using a 2 × 3 within-subjects design, we manipulated length-digit congruity, as previously noted, having the conditions of congruent (the number with more digits starts with a larger number, e.g., 4689 vs. 145) and incongruent number pairs (the number with more digits begins with a smaller number, e.g., 5372 vs. 859), and ratio of fillers (i.e., same-length pairs) within each block (25%, 50%, and 75% of fillers).

To analyze different-length pairs’ data, we conducted a frequentist and Bayesian repeated-measures ANOVA using JASP (version 0.14.1; JASP Team, 2020). For post-hoc analyses frequentist, Bonferroni-corrected pairwise comparisons were conducted, and therefore the corrected p-values for these comparisons are reported. Partial eta-squared values (η_p_^2^) were computed and reported as a measure of effect size for the ANOVA (Cohen, 1988; Lakens, 2013). Bayes factors for each effect were computed by comparing models that contain the effect to equivalent models without that effect. In this way, we provide evidence for the inclusion of each factor, and interactions, in the model (following standard notation BF_10_ quantifies support for the alternative hypothesis whereas BF_01_ quantifies support for the null hypothesis) (van den Bergh et al., 2020). Data visualization was made in R (version 4.1.1; R Core Team, 2021) using the *ggplot2* (version 3.3.5; Wickham, 2016) and *dplyr* (version 1.0.7; Wickham et al., 2021) packages. The same practices were used in the following experiments. The analyses, R scripts and data from this and the other experiments are available at OSF (https://osf.io/5mnu9/).

### Results

In Table 2 we present the mean response times and percentage of errors for each type of number pairs in each block condition. As expected, response times were considerably larger (about 200 ms) in the same-length condition than in the different-length condition. Similarly, errors ranged between 6.2 and 17.5% in the same-length conditions, being practically negligible in the different-length conditions (range 0.2–2%). The raw and processed data from this and the other experiments are fully available from https://osf.io/5mnu9/.

**Table 2:** Mean response times and percentage of error responses (in brackets) for the same-length pairs and for the length-digit congruent and incongruent trials in the different-length condition in Experiment 1

Block (%)	Same-length pairs		Different-length pairs		
	Three-digits	Four-digits	Congruent	Incongruent	Difference
25	865 (11.3)	860 (17.5)	624 (0.5)	633 (1)	9 (0.5)
50	899 (8.5)	916 (10.2)	691 (0.2)	702 (2)	11 (1.8)
75	915 (6.2)	935 (8.9)	743 (0.6)	761 (1.1)	18 (0.5)

Given that accuracy was at its ceiling in the different-length condition, only response times were analyzed. Prior to the analysis we excluded the incorrect responses and the correct responses briefer than 250 ms and larger than 1500 (less than 1.7% of the total data). Response times to correct responses were subject to an ANOVA, with the within-subjects factors congruity (congruent, incongruent) and filler ratio (25%, 50%, 75%).

The two-way ANOVA yielded main effects of congruity, *F*(1, 35) = 11.79, *p* = 0.002, ƞ_p_^2^ = 0.25, BF_10_ = 2.04, pointing to the existence of slower responses (about 10 ms) in the incongruent condition. There was also a significant effect of filler ratio, *F*(2, 70) = 90.48, *p* < 0.001, ƞ_p_^2^ = 0.72, BF_10_ = 2.13 × 10^39^. The data indicated an increase in response times as the proportion of fillers increases. Post-hoc Bonferroni-corrected analyses indicated differences between the three conditions (all *p*s < 0.001). Interestingly, there was no interaction between the two factors, *F*(2, 70) = 0.85, *p* = 0.43, ƞ_p_^2^ = 0.02, BF_10_ = 0.11.

### Discussion

The results indicated a main effect of filler ratio, and more importantly a main effect of congruity (although according to the Bayesian analysis the evidence is anecdotal), suggesting that when comparing natural numbers of different length, despite length being the relevant information, participants took into account the identity of the leftmost digits in such a way that when the shorter number began with a bigger number, this had a cost in reaching a decision on which multi-digit number was larger. This goes against a serial mechanism in which length is considered first, and leftmost digits are only compared in the case of length equality. On the contrary, results support the componential model in that two attributes, length and leftmost digits, influence performance in parallel. However, the results do not coincide completely with the assumptions of the model. According to previous studies, it was expected that the congruity effect would be modulated by the filler ratio, and no evidence of such interaction was found in the results: the congruity effect was similar in all three ratios conditions. A prediction of the componential model is that in an experimental set with a higher proportion of same-length numbers, more attention should be paid to the initial digits, and consequently a bigger impact of the leftmost digits should arise in the different-length pairs. According to the model, a cognitive control network exists that should be sensitive to variations in the proportion of fillers, and hence this should balance the relevance of length and initial digits in the decision process. This system should consider the relevance of each factor and adjust the weight of the connections between the length nodes and the initial digits comparison nodes accordingly (Huber et al., 2016). However, it seems that it was not sensitive to this factor in this case. There are several reasons for this (see “General discussion” for more details), but it can be argued that the absence of an interaction between congruity and filler ratio might be related to the procedure followed in the experiment: participants performed the three blocks in a single session and inter-blocks breaks were similar in duration, some seconds, to those in the intra-block session. All of this could in some way mask the proportions between the fillers and experimental pairs in each block. For instance, performing the 75% block immediately after the 50% block might not make a big difference for the cognitive control network, at least until a substantial number of items in the block were performed (e.g., Elston-Güttler et al., 2005). With the aim of testing this hypothesis and confirming the existence of a congruity effect that is not so clear according to the Bayesian analysis, we replicated Experiment 1 with a single modification: participants were requested to complete the three blocks in sessions separated by at least half an hour.

## Experiment 2

In this experiment, we replicated Experiment 1 with a single modification, with participants completing the three filler ratio blocks in three different sessions. This experiment was intended to replicate the presence of congruity effects and to test whether the absence of a modulation of the effect with the filler ratio found in Experiment 1 was related to running the task in a single session.

### Method

#### Participants

Forty undergraduate students from the University of Málaga took part voluntarily in this experiment. Their mean age was 22.70 (*SD* = 3.18, range 20–35 years, 3 males). All participants had a normal or corrected-to-normal vision and were naive regarding the purpose of the study. None of them have participated in Experiment 1.

#### Materials

These were the same as those employed in Experiment 1. Due to a software programming error 6 same-length pairs were unintentionally repeated in the 50% filler ratio condition, and thus instead of a ratio of 50% it was 51.5% (despite this, we will continue to refer to this condition as the 50% filler ratio block).

#### Procedure

There was only one difference regarding the procedure followed in Experiment 1: participants performed each block in different sessions. Sessions were separated by at least 30 min, although in most cases they were carried out on different days.

### Design and analysis

The same design and analysis as in Experiment 1 were followed.

### Results

Results are summarized in Table 3 and resemble those found in Experiment 1. The same-length condition was about 185 ms slower than the different-length condition. Similarly, errors ranged between 7.1 and 18.3% in the same-length conditions, being practically negligible in the different-length conditions (range: 0.05–1%).

**Table 3:** Mean response times and percentage of error responses (in brackets) for the same-length pairs and for the length-digit congruent and incongruent trials in the different-length condition in Experiment 2

Block (%)	Same-length pairs		Different-length pairs		
	Three-digits	Four-digits	Congruent	Incongruent	Difference
25	876 (11.6)	882 (18.3)	664 (0.3)	678 (0.3)	14 (0)
50	892 (9.2)	908 (12)	712 (0.7)	726 (1)	14 (0.3)
75	913 (7.1)	923 (8.7)	746 (0.05)	766 (0.9)	20 (0.85)

Due to the small number of errors in the different-length condition only response times were analyzed. Prior to the analysis, we excluded the incorrect responses and the correct responses briefer than 250 ms and larger than 1500 (less than 1.1% of the total data). Response times to correct responses were subject to an ANOVA, with the within-subjects factors congruity (congruent, incongruent) and filler ratio (25%, 50%, 75%).

The two-way ANOVA yielded effects of congruity, *F*(1, 39) = 43.75, *p* < 0.001, ƞ_p_^2^ = 0.53, BF_10_ = 4.34. Responses to incongruent pairs were about 16 ms slower than to congruent pairs. There was also a significant effect of filler ratio, *F*(2, 78) = 30.19, *p* < 0.001, ƞ_p_^2^ = 0.43, BF_10_ = 1.80 × 10^20^. The data indicated an increase in response times as the proportion of fillers increases. Post-hoc Bonferroni-corrected analyses indicated differences between the three conditions (all *p*s < 0.003). As in Experiment 1, the interaction between both factors was not significant, *F*(2, 78) = 0.9, *p* = 0.41, ƞ_p_^2^ = 0.02, BF_10_ = 0.08.

### Discussion

**Fig. 1:**
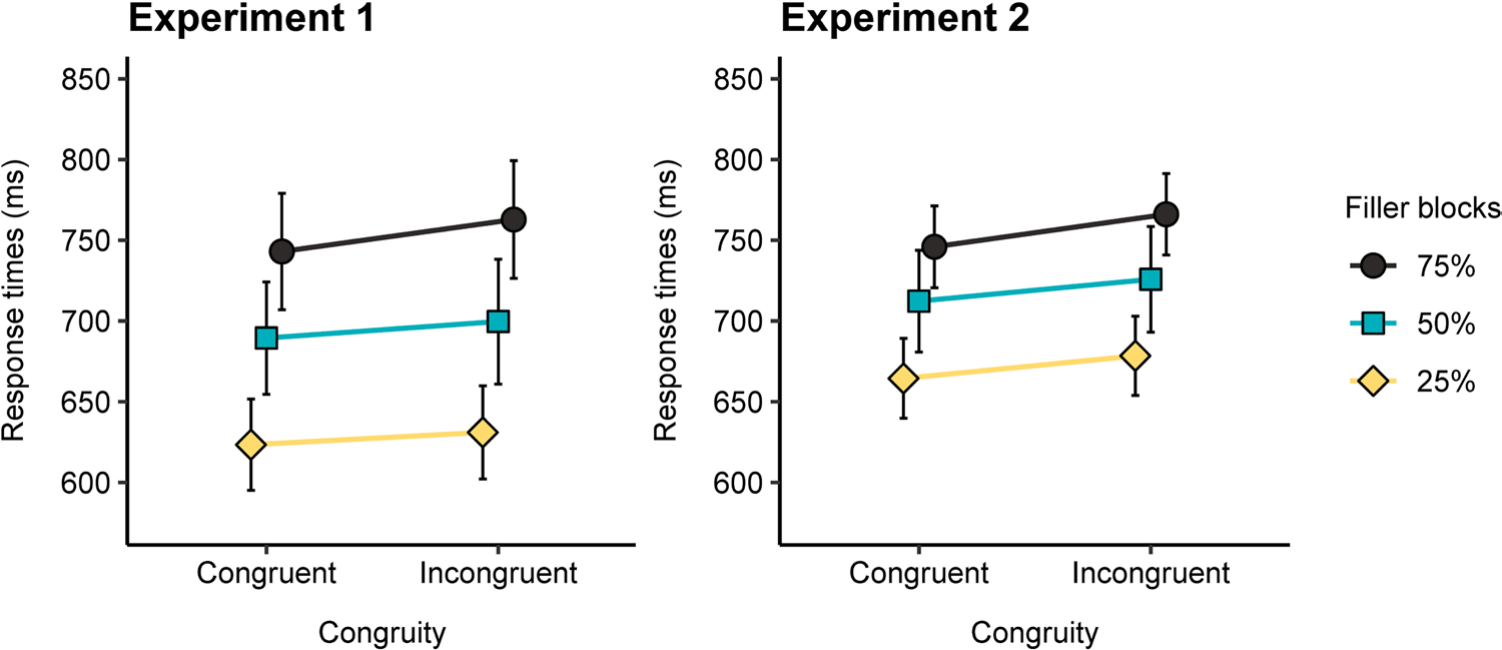
Mean response times for congruent and incongruent (different-length pairs) in the three filler ratio conditions in Experiments 1 and 2. The error bars indicate 95% confidence intervals

Results essentially reinforce ate the findings in Experiment 1: main effects of congruity and filler ratio, but no interaction between these factors (see Fig. 1). The findings, then, indicate the robustness of the congruity effect, but also that the influence of the leftmost digit when comparing different-length numbers is not modulated by the proportion of fillers. In an attempt to discount any lack of power in the design as the reason for the absence of the interaction between congruity and filler ratio, we re-ran the analysis pooling together the data from the different-length pairs of Experiments 1 and 2, and thus increasing the number of participants. Thus, the 2 × 3 × 2 mixed design included congruity and filler ratio as a within-subjects factor, and experiment as a between-subjects factor. The results were interesting. First, the analysis confirmed the main effects of congruity, *F*(1, 75) = 44.37, *p* < 0.001, ƞ_p_^2^ = 0.37, BF_10_ = 44.50, and filler ratio, *F*(2, 150) = 106.65, *p* < 0.001, ƞ_p_^2^ = 0.59, BF_10_ = 2.29 × 10^60^. There was an experiment by filler ratio interaction, *F*(2, 150) = 4.09, *p* = 0.019, ƞ_p_^2^ = 0.05, BF_10_ = 104.82, showing that the differences between 25%, 50% & 75% filler ratio was more sparse in Experiment 1 than in Experiment 2, likely due to more fatigue in the first experiment. The factor experiment was not significant, *F*(1, 75) = 1.53, *p* = 0.22, ƞ_p_^2^ = 0.02, BF_10_ = 0.72, and did not interact with any other effect (all *F*s < 1, BF_10_’s < 0.16). More importantly, the interaction between filler ratio and congruity approached the significance level, *F*(2, 78) = 2.44, *p* < 0.091, ƞ_p_^2^ = 0.031, BF_10_ = 0.07. This interaction was probably due to the existence of a bigger congruity effect in the 75% fillers condition (20 ms) compared to the 25% and 50% conditions (10 and 12 ms, respectively). Despite the interaction not being significant, we ran a Post-hoc Bonferroni-corrected analysis to explore whether the congruity effect was present in all ratios. The analysis confirmed this (all *p*s < 0.001).

The results of the combined analysis suggest for the first time that the congruity effect may be modulated by the filler ratio, as is predicted by the componential model. However, it should be noted that the effect only approached significance, and a considerable number of participants were required to achieve this effect. Additionally, the BF provided no evidence in support of the interaction^4^. Anyway, with the aim of increasing power, we decided in Experiment 3 to make the experimental conditions more extreme (e.g., Giner-Sorolla et al., 2019; Lakens & Caldwell, 2021), contrasting a 50% filler condition against a pure condition with 0% of fillers.

## Experiment 3

In Experiments 1 and 2, it was observed that the proportion of fillers (i.e., pairs with the same length) played at most a small role in the length-digit congruity effect. No matter how many fillers are included, the leftmost digits play a role in the task. The present experiment aimed to show whether the congruity effect appears even in the absence of fillers, that is, in a situation where only different-length numbers are presented; we could then compare this with a condition with 50% of same-length pairs.

Additionally, in the previous experiments the leftmost digits of the different-length pairs had a distance of 1, and it is well known that smaller distances produce smaller congruity effects (e.g., García-Orza et al., 2016, 2017; Kallai & Tzelgov, 2012). Using distance 1 was forced on us due to our wish to create congruent and incongruent pairs with exactly the same overall distance (see Table 1); in fact, such a requisite may be unnecessary, since overall distance effects have not been observed in studies that employed multi-digits with more than three digits (e.g., Meyerhoff et al., 2012). In this experiment, we relaxed this condition and in addition to pairs of digits in which the leftmost digits have a distance of 1, we created pairs that have a distance of 3 and 4, although we still tried to keep differences in overall distance to a minimum.

Therefore, we presented the participants a numerical comparison task in two blocks: pure and mixed. In the pure block, we included only pairs of multi-digit numbers of different lengths (3 vs. 4). The other block was the mixed one. In addition to different-length pairs (3 vs. 4), it contained same-length number pairs as fillers (50% of the total), half of which were 3 vs. 3 and 4 vs. 4 number pairs. In both blocks, different-length pairs were length-digit congruent (e.g., 4689 vs. 145) or length-digit incongruent (e.g., 5372 vs. 859). These numbers in the congruent and incongruent condition had either a distance of 1 (e.g., 2478 vs. 103 and 2764 vs. 389) or of 3–4 (e.g., 5598 vs. 145 and 5468 vs. 978) between the leftmost digits.

Finding congruity effects in the pure block, that is, in a condition where simply focusing on the amount of digits in each number is sufficient to solve the task, would suggest that paying attention to the first digits, despite being irrelevant, is mandatory and would reinforce a componential view. However, it would also suggest that the demands of the task are not managed properly by the cognitive control network. On the contrary, the absence of congruity effects in the pure condition, together with the lack of filler ratio × congruity effects in previous experiments, would show that only under extreme conditions (0% fillers) is the cognitive control network able to manage the task in such a way that only length is considered and leftmost digits are ignored.

Additionally, it is in principle expected that the size of the congruent effect would be larger in the distance 3–4 condition, as it is easier to detect large distances than small ones. This differential effect would indicate that the processing of initial numbers involves deep processing of quantity, whereas no differences in the size of the congruent effect might suggest that these are simply categorized as big or small (e.g., see Kallai & Tzelgov, 2012; García-Orza et al., 2016; Tzelgov et al., 1992).

### Method

#### Participants

Twenty-six people took part voluntarily in this experiment. Their mean age was 31.27 (*SD* = 10.53, range: 17–51 years, 11 males). All participants had a normal or corrected-to-normal vision and were naive regarding the purpose of the study. One further participant did the experiment but was eliminated as she committed more than 20% errors.

#### Materials

As in previous experiments, here we included four types of pairs of multi-digit numbers, two types consisting of different-length numbers (comparing three- with four-digit numbers) and two types of trials of same-length numbers (comparing pairs of three- or pairs of four-digit numbers). Within different-length pairs, 40 were length-digit congruent pairs, where the leftmost digit of the four-digit number was larger than the initial digit of the three-digit number. The other different-length pairs included 40 incongruent pairs, in which the leftmost digit of the four-digit number was smaller than the leftmost digit of the three-digit number (e.g., 5372 vs. 859). Moreover, the distance between the leftmost digit was 1 in half of the congruent and incongruent pairs (they were taken from the stimuli employed in Experiments 1 and 2), whereas it was 3 or 4 in the other half of the stimuli (see Table 4). Overall distance between congruent and incongruent pairs was exactly the same for distance 1 pairs, and slightly different for the congruent pairs in the distance 3 condition (congruent: *M* = 4753.75, *SD* = 541.70; incongruent: *M* = 4780.30, *SD* = 471.25), *t*(19) = 0.14, *p* = 0.89.

**Table 4:** An example of the experimental stimuli in Experiment 3

Pair type	Pair sample		Distance	
	Bigger number	Smaller number	Overall	Leftmost digit
Length-digit Congruent	5324	121	5203	4
	2358	104	2254	1
Length-digit Incongruent	5674	978	4696	4
	2641	387	2254	1

To be used as fillers, 80 same-length pairs (40 three-digit pairs and 40 four-digit pairs) were created. These stimuli maintained the same characteristics as the different-length pairs in regard to the distance between the leftmost digits, so in half of the three- and four-digit pairs, the distance was 1 and in the other half it was 3 or 4.

Two blocks were created. The pure block included exclusively congruent and incongruent pairs of different lengths, whereas the mixed block added to the different-length pairs those with the same length in a 50% ratio. An example of the stimuli is presented in Table 4, and the complete list of stimuli is provided in Appendix B.

#### Procedure

Seven of the participants were tested in the Numerical Cognition Lab at the Faculty of Psychology and Speech Therapy under the supervision of two researchers. Due to the COVID-19 pandemic restrictions, the rest of the participants performed the test individually in their homes, in a controlled and calm environment, overseen by one of the researchers. The same presentation procedure employed in previous experiments was followed. For all participants, the experiment was run on a 14-inch screen-size laptop.

The experiment consisted of two blocklists that were presented to participants in successive order. In the mixed block, a sum of 320 trials was run: 40 trials in four existing conditions (congruent, incongruent, same-length three-digit numbers, and same-length four-digit numbers). In half of the numbers in the pairs, the leftmost digits maintained a distance of 1, and in the other half a distance of 3–4. Each trial was presented twice, once with the numerically larger number on the right side, and once with the larger number on the left side. The duration of the mixed block was approximately 13–14 min. In the pure block, only the 160 different-length congruent and incongruent trials employed in the mixed block were presented. Half of the participants carried out the pure block first, then the mixed block; the other half of the participants performed the experiment in the reverse order. The order of item presentation in each block was randomized.

### Design and analysis

In this third experiment, we added a new within-subjects factor – the distance between the leftmost digits of the numbers to be compared. In specific, using a 2 × 2 × 2 within-subjects design, we manipulated the congruity (congruent and incongruent number pairs), ratio of fillers in each block (0% in the pure block and 50% in the mixed block), and distance between the leftmost digits (distance 1 and distance 3–4).

### Results

Mean correct response times and the proportion of errors for each condition are reported in Table 5. Participants were almost 150 ms slower in the same length than in the different-length condition. Errors were also considerably higher in the former condition (range: 7.6–11.5%) than in the latter (range: 0.2–2.1%). The overall proportion of errors was small in the different-length condition, and hence, as in the previous experiments, errors were not analyzed.

**Table 5:** Mean response times and percentage of error responses (in brackets) for the same-length pairs and for the length-digit congruent and incongruent trials in distances 1 and 3–4 in the different-length condition in Experiment 3

Block	Same-length pairs		Different-length pairs					
			Distance 1			Distance 3–4		
	Three-digits	Four-digits	Congruent	Incongruent	Difference	Congruent	Incongruent	Difference
Mixed	650 (7.6)	696 (11.5)	499 (0.9)	518 (1.1)	19 (0.2)	492 (0.2)	522 (1.3)	30 (1.1)
Pure	–	–	406 (1.7)	408 (1.4)	2 (− 0.3)	403 (1.8)	410 (2.1)	7 (0.3)

For the analysis, within-subjects ANOVAs with the factors congruity (congruent, incongruent), distance (1, 3–4) and block (pure, mixed) were conducted on mean correct RTs of different-length pairs. The analysis of the response times showed significant main effects of block, *F*(1, 25) = 132.14, *p* < 0.001, η_p_^2^ = 0.84, BF_10_ = 6.45 × 10^57^, indicating that in the mixed block participants were, about 100 ms, slower than in the pure condition. There were also significant differences in congruity, *F*(1, 25) = 33.06, *p* < 0.001, η_p_^2^ = 0.57, BF_10_ = 77.66, with congruent trials being about 15 ms faster than incongruent trials. Moreover, the interaction of block and congruity yielded significant effects, *F*(1, 25) = 20.62, *p* < 0.001, η_p_^2^ = 0.45, BF_10_ = 3.63, caused by a bigger length-digit congruity effect in the mixed (24 ms) than in the pure condition (5 ms). Post-hoc analyses indicated that the congruity effect was significant in the first condition but not in the pure condition (*p* < 0.001 and *p* = 0.99, respectively).

No significant main effects of distance between the leftmost digits of the numbers to be compared were obtained, *F*(1, 25) = 0.24, *p* = 0.63, η_p_^2^ = 0.01, BF_10_ = 0.15. The interaction of block with distance did not yield significant results *F*(1, 25) = 0.14, *p* = 0.71, η_p_^2^ = 0.01, BF_10_ = 0.21, and neither did the interaction of congruity with distance, *F*(1, 25) = 1.99, *p* = 0.17, η_p_^2^ = 0.07, BF_10_ = 0.29. Likewise, no significant interaction between block, congruity and distance was found, *F*(1, 25) = 0.46, *p* = 0.50, η_p_^2^ = 0.02, BF_10_ = 0.30.

### Discussion

**Fig. 2:**
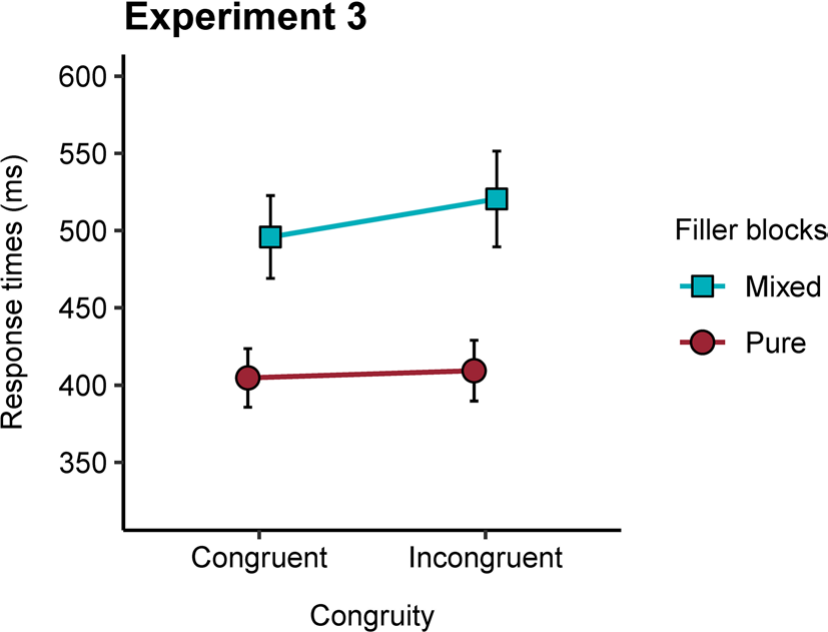
Mean response times for congruent and incongruent (different-length pairs) in the two filler ratio conditions in Experiment 3. The error bars indicate 95% confidence intervals

The results of this experiment provide the first evidence of congruity being sensitive to the proportion of fillers. When the comparison task included same-length numbers the effect was about 20 ms, replicating the results of previous experiments. In contrast, it was small, 4.6 ms, and non-significant when only different-length pairs were presented (see Fig. 2). Although the descriptives in previous experiments supported this interaction, no significant effects were found, and even the Bayesian analyses provided substantive support for the null hypothesis. An extreme manipulation was needed to find a context-related modulation of the congruity effect. This suggests that the cognitive control system is only slightly sensitive to strong manipulations of the context.

The lack of a congruity effect in the pure condition is interesting. It implies that our processing mechanisms avoid the comparison of the leftmost digits in a context without same-length numbers.

Finally, we found no evidence of a modulation of the congruity effect with the distance of the leftmost digits. According to previous studies (e.g., Kallai et al., 2012; García-Orza et al., 2017), it was expected that the congruity effects would increase when the distance between the leftmost digits was bigger. Our data do not provide evidence in this direction, possibly suggesting that the analysis of these numbers is shallow and the congruity effect is simply caused by tagging the leftmost digits as “bigger” or “smaller” (e.g., see Tzelgov et al., 1992).

## General discussion

Understanding how our mind builds up a numerical value and compares multi-digit numbers has been the focus of recent research in numerical cognition. Data suggests that comparing multi-digit numbers usually involves combining different types of information in parallel. This evidence has been nicely accounted for in Huber and colleagues’ componential model (see Huber et al., 2016). With the aim of testing this model, we conducted three experiments which explored whether the length is the only attribute considered in deciding which number string represents a larger number or whether other attributes, like the leftmost digit of each number, contribute to this process. Specifically, we investigated for the first time the simultaneous comparison of natural numbers that differ in length, a task that despite being a common practice in daily life, has been almost entirely neglected in numerical cognition research. Participants were asked to compare length-digit congruent number pairs (e.g., 2384 vs. 107) and length-digit incongruent number pairs (e.g., 2675 vs. 398) under different proportions of same/different-length number pairs. The main findings of the three experiments are: (i) Length was processed fast and accurately to decide whether natural numbers were bigger or smaller. This was shown simply by observing the accuracy and speed of responses in different-length pairs (percentage of errors smaller than 2% and RTs faster than 750 ms) and same-length pairs (percentage of errors around 10% and RTs slower than 850 ms); (ii) Across three experiments a length-digit congruity effect was found, this indicating an influence of the leftmost digits in the comparison task, so that when the leftmost digit in a three-digit length number was bigger than the leftmost digit in a four-digit length number, a significant increase in RTs was observed; (iii) The length-digit congruity effect was modulated, although not strongly, by the same/different-length pairs ratio; it was only when the manipulation was extreme (50% vs. 0%), as in Experiment 3, that a difference in the size of the congruity effect with the proportion of fillers arose. In the following, we discuss in more depth the theoretical implication of these findings.

First, the percentages of errors and RTs were considerably smaller in the different-length pairs than in the same-length pairs comparison. This suggests that length is easily computed to solve the task when multi-digits differ in the number of digits. However, on top of this, the finding of a length-digit congruity effect indicates that participants could not avoid comparing the leftmost digits of each number despite the fact this information was not useful when faced with pairs of different lengths. In other words, the difference in length between the three-digit length numbers and the four-digit length numbers was not the only information involved when comparing these numbers in lists that also include same-length numbers. It seems that the comparison process does not follow a serial process by which, first, the length is compared, and then, only if it is the same for both numbers, the leftmost digits are compared in a second step. On the contrary, both length and the identity of the leftmost digits are attributes that were weighted in the comparison process. The length-digit congruity effect supports the existence of a parallel and componential process in line with the prediction of Huber’s componential model (Huber et al., 2016). This computational model distinguished, in the input layer, dedicated nodes for processing the magnitude of digits and the length of multi-digits. This information is contrasted in the comparison layer and different weights are assigned to each type of information (length and leftmost digits identity) according to the nature of the task. In our case specifically, since participants are faced with a comparison task with natural numbers, more weight is assigned to “number of digits” (i.e., length) comparison nodes and less to the node that compares the leftmost digits. The congruity effect reported in our three experiments comparing different-length natural numbers provides the first evidence in support of a parallel and interactive process in which length has a dominant role, but the information of the leftmost digit is also considered. This is exactly what is predicted by Huber’s et al., (2016) componential model.

Second, an additional aim of our experiments was to verify another assumption of the componential model: that the multiple attributes that are processed during the task are managed by a cognitive control network. This network, implemented as an error detection network, pays attention to task demands and context, and modifies the weights of the attributes to solve the task efficiently (Huber et al., 2016). The computational model has been successful in simulating the results of previous studies that have manipulated the stimulus set when exploring the unit-decade compatibility effect. These studies have shown that the unit-decade compatibility effect increases when the proportion of pairs of the same decade were augmented (Huber et al., 2013; Macizo & Herrera, 2011). In a context with high ratios of same-decade pairs, the cognitive control mechanism should increase the focus on the units to solve the comparison task, and this is implemented in the model by increasing the weight of the node corresponding to the units. Thus, when faced with an incompatible pair, despite differing in the decades, an influence of the units is observed (see Huber et al., 2016). In our experiments, by manipulating the ratio between same- and different-length numbers, we expected to find signs of the cognitive control mechanism proposed by the componential model: a bigger congruity effect when more same-length pairs are included. In this case, as the values of the leftmost digits are the more relevant information to solve those pairs; it is expected that the cognitive control network intervenes to add more weight to the unit that compares the leftmost digits than to the unit that compares “number of digits”. Evidence from Experiments 1 and 2 did not support a strong modulation of the congruity effect by the same-/different-length ratio. Only when pulling together data from both experiments was a trend towards significance found (*p* = 0.091, but there was even support for the null hypothesis in the Bayesian analysis BF_01_ = 14.28): the congruity effect in the 75% filler ratio block rose to 20 ms from the 10 ms and 12 ms that were found, respectively, in the 25% and 50% conditions. The absence of effects could be related to an insufficient number of participants, but it seems more clearly related to the small size of the effect and the soft manipulation of the filler ratio factor. In fact, a stronger manipulation of the filler ratio in Experiment 3 allowed us to find a significant modulation of the congruity effect. There was a robust congruity effect, about 20 ms, when the proportion of same-length numbers was 50% and this was absent when the participants were presented only with different-length numbers, and hence, where only length was needed to solve all the pairs in the task.

The moderate lack of sensitivity of the congruity effect to the filler proportions suggests that the cognitive control mechanism finds it difficult to assign zero relevance to the leftmost digit comparison node and full relevance to the length comparison node at least with ratios as small as 25%. It is only when blocks without different-length numbers that the relevance of the leftmost digits seems to disappear. This reduced sensitivity of the cognitive control mechanism to the same/different ratio may arise from the automaticity of leftmost digit comparison. Number comparison is almost an unavoidable process, and this is not surprising in light of extant research. The unit-decade compatibility effect already showed that our system compares digits (units) even though they are not relevant for the decision of the task (Nuerk et al., 2001); comparison effects have also been found in physical-size comparison tasks with numbers (e.g., Tzelgov et al., 1992) and even when numbers were interspersed between letters (García-Orza et al., 2016). Moreover, there is evidence, for example, that when presenting with two-digit numbers like 74, a comparison between 7 and 4 occurs, an intra-number comparison takes place (Nuerk, Moeller, et al., 2011; Nuerk, Willmes, et al., 2011).

It is also interesting that the manipulation of the numerical distance between the leftmost digits was not relevant in the task. In Experiment 3 no difference in the size of the congruity effect was found between pairs that have a distance between the leftmost digits of 3–4 (e.g., 5598 vs. 145 and 5468 vs. 978) and those that have a distance of 1 (e.g., 2478 vs. 103 and 2764 vs. 389). The absence of congruity × size effect suggests that a decision is reached before a deep analysis of the number's meaning is performed. It seems that the length-digit effect arises by tagging the leftmost digits simply as “smaller” or “bigger” without reaching a more detailed numerical representation (see García-Orza et al., 2017; Kallai & Tzelgov, 2012).

An important thing to note is that, although no differences in overall distance between the congruent and incongruent number pairs existed, there were differences in terms of ratio. In fact, in Experiments 1 and 2 and Experiment 3 the correlation between congruity and ratio was 0.90 and 0.88, respectively (both *ps* > 0.001). Although recent research has cast doubt on the role of ratio in comparing symbolic numbers (e.g., Krajcsi et al., 2018; Lyons et al., 2015; Marinova et al., 2021) and the evidence of a decomposed processing of multi-digits is overwhelming (e.g., García-Orza & Damas, 2011; Huber et al., 2016; Meyerhoff et al., 2012; Nuerk, Moeller, et al., 2011; Nuerk, Willmes, et al., 2011; Poltrock & Schwartz, 1984), in this paragraph we explore whether ratio may underlie congruity effects in our experiments. Our data does not support this view for several reasons. First, a ratio effect cannot explain the absence of congruity effects in the pure condition of Experiment 3, in which the same different-length pairs as those in the mixed condition are used. Since ratio models assume that ratio computing is automatic, then differences in the proportion of same/different-length stimuli should not affect the ratio mechanisms that are based on overall number value, and hence, an effect should be observed in both conditions. Second, in Experiment 3, the distance between the left-most digits was manipulated to explore whether the processing of these numbers was precise enough (i.e., distance was computed) or if they were simply labeled as small or big. This manipulation created significant ratio differences between the congruent and incongruent pairs in those pairs with a leftmost digit difference of 3–4 (*M* = 30.11, *SD* = 4.48) and those with a leftmost digit difference of 1 (*M* = 5.70, *SD* = 4.24; *t*(38) = 14.7, *p* < 0.001), however, these differences in ratio did not translate into differences in the size of the congruity effect for these pairs of stimuli (*p* > 0.17, BF_10_ < 0.30). Third, the previously commented lack of left-most digit distance effect on congruity has additional implications for our debate. We have argued that this result can be interpreted as evidence of a surface processing of the left-most digits as “bigger” or “smaller”. In this frame, if the exact distance between the two left-most digits is not computed, then it seems quite implausible that the ratio between both quantities might drive the congruity effect. Finally, we run additional multilevel regression analyses to separate the effects of congruity and ratio in the pooled data of Experiments 1 & 2 and in Experiment 3. The analyses are described in detail in Appendix C^5^. In Experiments 1 and 2, effects of fillers ratio and congruity were found whereas the ratio was not significant. The rest of the contrasts and interactions were not significant either. Regarding Experiment 3, the analysis again revealed significant effects of congruity, an interaction between congruity and fillers ratio and a significant effect of ratio. Altogether, these new analyses make it unlikely that the congruity effect was due to differences in ratio. Considering all the evidence described in this paragraph, it seems that the congruity effect we found is a genuine indication of the influence of the left-most digits when comparing different-length numbers.

In sum, our findings are in line with most of the proposals of the componential model of multi-digit processing (Huber et al., 2016). Length-digit comparison occurs as the model predicted: multiple attributes of the numbers seem to be processed by a different network of layers and nodes working in parallel and are weighted by the relevance of the task involved. Results on the relative insensitivity of the cognitive control mechanism in the context can also be accommodated within the model, by simply assuming a limited range in the weight assigned to the number of digits node and the leftmost digits when faced with different-length numbers. There is, however, an aspect that does not fit exactly with the model: it assumes that the digits composing the multi-digits are compared in accordance with their corresponding place-value (i.e., thousands vs. thousands, hundreds vs. hundreds…) and thus it proposes the existence of dedicated nodes to compare thousands, hundreds, etc. Yet the evidence suggests, on the contrary, that place-value is computed later and the existence of a different number of digits in each number pair creates an asymmetry: when faced with different-length numbers it seems that our participants compared initial numbers starting from the left of the multi-digit although they do not have the same place-value (thousands vs. hundreds). For a proper comparison between digits with the same place value we need to know how many numbers are included in the string. In light of our data, it seems that before getting that information participants compared the leftmost digits. In other words, the comparison between numbers started well before differences in length were considered and place-value was properly assigned. This conclusion, however, should be taken with caution due to the way the experimental stimuli were created; we have to recall that with the aim of avoiding compatibility effects between numbers in the different position, the three first digits of one of the multi-digits in the pair all had a bigger value than the three digits in the other multi-digit of the pair. This might have generated a sense of “bigger” or “smaller” associated with one of the numbers in the pairs, instead of there being an activation of the leftmost initial digits, as we assumed when explaining the congruity effect.

Finally, although it is beyond the scope of the present study, research on multi-digit numbers has to deal with the existence of spatial-numerical associations in the comparison task: more digits involve more elements and a perceptually larger item. This may favor reaching a decision on which is the bigger number. However, further research has to determine whether, to identify the larger number, participants compute the precise number of digits in each multi-digit, that is, 4 and 3, and compare them, or if they simply employ an approximate perceptual strategy of detecting physically bigger stimuli (see Cipora et al., 2018, for a useful taxonomy on spatial numerical associations that distinguish between approximate and exact associations). At present, whichever one of these mechanisms is in operation, the evidence indicates that this type of information is processed fast and accurately.

## Conclusion

In the current study, we were able to empirically demonstrate the theorized function of Huber’s componential model (Huber et al., 2016). When comparing multi-digits, several number attributes, such as the length and value of the leftmost digits of each multi-digit, are analyzed simultaneously. The finding of longer response times when shorter numbers start with a larger number (than when shorter numbers start with a smaller number) revealed that length is not the only information taken into account when comparing different-length number pairs; the leftmost digits are also considered. It seems, then, that commercial strategies correctly assume that length is prioritized when using offers like “was $1000, now only $999”, but we should not forget that buyers also compare the 9 with the 1, and thus in some part of their cognitive system 999 is considered bigger than 1000, at least for a while.

## Appendix A

Complete list of experimental stimuli (different-length pairs) in Experiments 1 & 2 together with the overall distance between the numbers in the pairs and the distance between the leftmost digits in each pair.

**Table:**

Congruent pairs				Incongruent pairs			
Pair		Distance		Pair		Distance	
		Overall	Leftmost digit			Overall	Leftmost digit
104	2358	2254	1	387	2641	2254	1
130	2456	2326	1	384	2710	2326	1
103	2478	2375	1	389	2764	2375	1
215	3470	3255	1	496	3751	3255	1
216	3479	3263	1	489	3752	3263	1
201	3465	3264	1	486	3750	3264	1
241	3569	3328	1	497	3825	3328	1
301	4526	4225	1	592	4817	4225	1
320	4561	4241	1	596	4837	4241	1
316	4582	4266	1	597	4863	4266	1
402	5163	4761	1	678	5439	4761	1
406	5179	4773	1	698	5471	4773	1
403	5187	4784	1	689	5473	4784	1
502	6134	5632	1	749	6381	5632	1
503	6178	5675	1	753	6428	5675	1
501	6248	5747	1	784	6531	5747	1
603	7158	6555	1	851	7406	6555	1
610	7253	6643	1	861	7504	6643	1
613	7285	6672	1	869	7541	6672	1
618	7293	6675	1	864	7539	6675	1
704	8159	7455	1	951	8406	7455	1
710	8243	7533	1	971	8504	7533	1
712	8349	7637	1	973	8610	7637	1
724	8365	7641	1	972	8613	7641	1

## Appendix B

Complete list of experimental stimuli (different-length pairs) in Experiment 3 together with the overall distance between the numbers in the pairs and the distance between the leftmost digits in each pair.

**Table:**

Congruent pairs				Incongruent pairs			
Pair		Distance		Pair		Distance	
		Overall	Leftmost digit			Overall	Leftmost digit
5324	121	5203	4	5674	978	4696	4
5483	132	5351	4	5423	965	4458	4
5763	165	5598	4	5671	978	4693	4
5674	152	5522	4	5742	986	4756	4
5486	125	5361	4	5357	958	4399	4
5598	145	5453	4	5468	978	4490	4
5689	156	5533	4	5579	968	4611	4
4567	123	4444	3	5431	897	4534	3
4467	134	4333	3	5462	857	4605	3
4689	145	4544	3	5372	859	4513	3
4897	178	4719	3	5263	879	4384	3
4478	135	4343	3	5563	887	4676	3
4597	124	4473	3	5654	876	4778	3
4985	164	4821	3	5663	897	4766	3
4254	102	4152	3	5135	846	4289	3
4365	124	4241	3	5246	856	4390	3
4245	113	4132	3	6354	947	5407	3
4365	125	4240	3	6475	968	5507	3
4476	136	4340	3	6876	998	5878	3
4397	125	4272	3	6763	987	5776	3
2358	104	2254	1	2641	387	2254	1
2456	130	2326	1	2710	384	2326	1
2478	103	2375	1	2764	389	2375	1
4526	301	4225	1	4817	592	4225	1
4561	320	4241	1	4837	596	4241	1
4582	316	4266	1	4863	597	4266	1
5163	402	4761	1	5439	678	4761	1
5179	406	4773	1	5471	698	4773	1
5187	403	4784	1	5473	689	4784	1
6134	502	5632	1	6381	749	5632	1
6178	503	5675	1	6428	753	5675	1
6248	501	5747	1	6531	784	5747	1
7158	603	6555	1	7406	851	6555	1
7253	610	6643	1	7504	861	6643	1
7285	613	6672	1	7541	869	6672	1
7293	618	6675	1	7539	864	6675	1
8159	704	7455	1	8406	951	7455	1
8243	710	7533	1	8504	971	7533	1
8349	712	7637	1	8610	973	7637	1
8365	724	7641	1	8613	972	7641	1

## Appendix C

### Linear mixed-effects models exploring the role of ratio in Experiments 1 and 2 pooled data, and Experiment 3

Linear mixed-effects (LME) models fitting by maximum likelihood (ML) were performed on latency data in R using the *lme4 *package (version 1.1–27.1; Bates et al., 2015). Models included congruity, fillers ratio and ratio and their interaction as fixed factors. Contrast coding was used for congruity and fillers ratio, while the ratio was zero-centered (e.g., Schad et al., 2020). Following the recommendations by Barr et al., (2013) and the practices in previous studies in the field (e.g., Huber et al., 2019), we employed the maximal random structure in terms of by-subject and by-item intercepts and slopes that converged for each experiment: RT ~ Congruity × Fillers ratio × Ratio + (1 + Fillers ratio|Subject) + (1|Item). This maximal random structure was identified using the *buildmer* package (version 2.1; Voeten, 2021, see also Voeten, 2019). The R code with the identification procedure for the maximal random structure, the best fitting models and their outputs is accessible https://osf.io/5mnu9/. The significance of the fixed effects was calculated with type III (Satterthwaite method) model comparisons using the mixed function in the *afex* package (version 1.0–1; Singmann et al., 2015), and (Bonferroni-corrected) post-hoc pairwise comparisons were performed using the *emmeans *package (version 1.7.0; Lenth et al., 2018). The mixed model analysis for the pooled data of Experiments 1 & 2 revealed a significant effect of fillers ratio, *F*(2, 165.76) = 62.74, *p* < 0.001. There was a main effect of congruity, *F*(1, 21,680.95) = 6.73, *p* = 0.009, with shorter response times when comparing different-length numbers in the congruent condition than in the incongruent condition (Δ = 16 ms). The interaction between congruity and fillers ratio was non-significant, *F*(2, 21,675.88) = 0.43, *p* = 0.65. These results obtained here follow the main results obtained in the previous analyses in Experiments 1 and 2. Ratio was not relevant, the only contrast that approached significance was the interaction between ratio and congruity, *F*(1, 21,677.95) = 3.12, *p* = 0.08, and the rest of the contrasts were far from significance (all *p*s > 0.69). Regarding Experiment 3, the mixed model analysis revealed significant effects of congruity, *F*(1, 8289.64) = 6.52, *p* = 0.011, fillers ratio, *F*(1, 36.64) = 122.49, *p* < 0.001, and an interaction between congruity and fillers ratio, *F*(1, 8262.77) = 4.97, *p* = 0.026. Post-hoc analyses showed no differences between congruity conditions in the pure block (0% fillers), *z* = 0.23, *p* > 0.99, but faster responses to congruent than to incongruent pairs in the mixed block condition, *z* = 3.41, *p* = 0.003. In this experiment, the main effect of ratio was significant, *F*(1, 8277.52) = 10.42, *p* = 0.001, showing an increase in RTs as ratio increases. The remaining interactions were all non-significant, all *p*s > 0.10.

## Footnotes

1 We have preferred to use the term “length-digit congruity effect” instead of “string length congruity effect” employed by Huber et al. (2014) when exploring decimals. In this way, we seek to emphasise the fact that length is the driving factor in the resolution process, whereas the identity of the leftmost digit is the irrelevant factor. This contrasts with the case of decimals, in which numbers’ identity is the driving force in the decision, and the length of the string creates the incongruity.

2 Although this result is also compatible with a holistic model, Poltrock and Schwartz (1984) also discarded a holistic model, because when they compared pairs of numbers that had two differing numbers in the same position, and which thus differed in overall distance, no differences were found

3 It should be borne in mind that the fillers ratio and the number of trials in each filler ratio condition were correlated, so the finding of a filler ratio effect can be confounded with fatigue effects. In any case, the main effects of the filler ratio are of no theoretical significance in terms of the aims of this study, and thus will not be interpreted in this research.

4 Despite having a considerable number of participants pooling together the data from both experiments, the absence of an interaction may still be due to an insufficient number of participants. To check this, we ran a sensitivity analysis using the Shiny app for the *ANOVA_power* function of the *Superpower* R package (https://arcstats.io/shiny/anova-power/, Lakens & Caldwell, 2021) with *N* = 76, *SD* = 100 and a within-factors correlation *r* = 0.75. A parametric manipulation of the interaction effects expected in the population was run, taking a fixed effect of fillers ratio of 25 ms per condition and a linear increase (× 2 from condition to condition) of the congruity effect at each level of fillers ratio. We simulated differences starting at 5, 10 and 20 ms in the 25% fillers ratio, respectively, for small, medium and large effect sizes. Results indicated that our study seems well powered (99%) to find a strong effect (ƞ^2^_p_ = 0.17), but somehow low powered to find medium (66%, ƞ^2^_p_ = 0.06) and small effects (20%, ƞ^2^_p_ = 0.02). This suggests the interaction effect we are studying, if indeed it exists, is medium to low at best (the sensitivity analyses can also be seen at https://osf.io/5mnu9/).

5 As pointed out by a reviewer, these analyses should be taken with caution due to two different factors: (a) the high correlation between ratio and congruity; (b) the indeterminacy of the models supporting ratio or ratio-like factors (e.g., see the Discrete Semantic System, Krajcsi et al., 2016) regarding the specific regressors to include in the analysis.
